# Diversity and distribution of microbial communities in floral nectar of two night-blooming plants of the Sonoran Desert

**DOI:** 10.1371/journal.pone.0225309

**Published:** 2019-12-12

**Authors:** Martin von Arx, Autumn Moore, Goggy Davidowitz, A. Elizabeth Arnold

**Affiliations:** 1 Department of Entomology, The University of Arizona, Tucson, AZ, United States of America; 2 School of Plant Sciences and Department of Ecology and Evolutionary Biology, The University of Arizona, Tucson, AZ, United States of America; Indian Institute of Science, INDIA

## Abstract

Nectar-inhabiting microbes are increasingly appreciated as important components of plant-pollinator interactions. We quantified the incidence, abundance, diversity, and composition of bacterial and fungal communities in floral nectar of two night-blooming plants of the Sonoran Desert over the course of a flowering season: *Datura wrightii* (Solanaceae), which is pollinated by hawkmoths, and *Agave palmeri* (Agavaceae), which is pollinated by bats but visited by hawkmoths that forage for nectar. We examined the relevance of growing environment (greenhouse vs. field), time (before and after anthesis), season (from early to late in the flowering season), and flower visitors (excluded via mesh sleeves or allowed to visit flowers naturally) in shaping microbial assemblages in nectar. We isolated and identified bacteria and fungi from >300 nectar samples to estimate richness and taxonomic composition. Our results show that microbes were common in *D*. *wrightii* and *A*. *palmeri* nectar in the greenhouse but more so in field environments, both before and especially after anthesis. Bacteria were isolated more frequently than fungi. The abundance of microbes in nectar of *D*. *wrightii* peaked near the middle of the flowering season. Microbes generally were more abundant as time for floral visitation increased. The composition of bacterial and especially fungal communities differed significantly between nectars of *D*. *wrightii* and *A*. *palmeri*, opening the door to future studies examining their functional roles in shaping nectar chemistry, attractiveness, and pollinator specialization.

## Introduction

Nectar-inhabiting microbes are increasingly appreciated as important components in many plant-pollinator systems [1,2]. Bacteria and fungi, including both yeasts and filamentous fungi, have been identified in nectar of diverse plant species [3–4]. They can influence nectar chemistry, volatiles, pollen germination, floral attractiveness, and nutritional quality for pollinators [2, 5–7]. Diverse studies have identified microbial communities in nectar in various temperate and tropical plants [1–8], but relatively little is known about nectar-inhabiting microbes in desert flowers.

In areas such as the Sonoran Desert, known for richness both of flowering plants and pollinators, a rich history of studies has characterized plant-pollinator associations [9–11]. However, to our knowledge only two studies have examined the diversity of nectar-inhabiting microbes in the Sonoran Desert bioregion: [12] examined nectar of saguaro cactus (*Carnegiea gigantea*) and cultivars of *Citrus*, and [13] examined nectar from cultivars of cotton in field- and greenhouse settings, as well as saguaro, prickly pear cactus (*Opuntia*), and *Citrus*. These studies showed that bacteria (*Staphylococcus*, other Gram-positive bacteria, and some Gram-negative strains) were present but not highly diverse or abundant in nectar of saguaro [12, 13]. In *Citrus* they recorded bacteria, but only rarely, with the sum of their work highlighting infrequent infections by Gram-negative bacteria, one fungus, and one actinomycete [12, 13]. These studies did not identify nectar-inhabiting microbes to species nor compare and contrast them among host plants, evaluate their seasonality, or explore their abundance before and after anthesis. Thus little is known regarding the microbial diversity in nectar of Sonoran Desert plants.

Understanding the diversity and distributions of nectar-inhabiting microbes is a key step in understanding the interactions that shape the diverse biotic communities of the arid southwest, which are threatened increasingly by human activity at local scales [9, 10] as well as climate change more broadly [14]. The aims of this study were to characterize the diversity and distributions of microbes associated with nectar of two iconic plants of the Sonoran Desert region, where previous studies of nectar microbiomes have been limited in scope. Specifically, we quantified the frequency, abundance, diversity, and composition of bacterial and fungal communities in floral nectar of two species of night-blooming plants: *Datura wrightii* (Solanaceae), which is pollinated by hawkmoths [15], and *Agave palmeri* (Agavaceae), which is pollinated by bats but visited by hawkmoths that forage for nectar [15–17]. These two species differ in their nectar composition, concentration, floral display, flower longevity, and flowering phenology [15], yet are both a major source of nectar for the hawkmoth community of the southwest [16, 17]. Here we describe the relevance of growing environment (greenhouse vs. field), time (before and after anthesis), season (from early to late in the flowering season), and flower visitors (excluded via mesh sleeves or allowed to visit flowers naturally) in shaping microbial communities in nectar. Our work complements the growing literature regarding nectar-inhabiting microbes in other biotic communities [1–8, 18–24] while also addressing key gaps in existing knowledge of the biodiversity and biotic interactions between plants and other organisms in the Sonoran Desert region [9, 10].

## Materials and methods

Nectar was collected from *D*. *wrightii* and *A*. *palmeri* flowers on plants in field and greenhouse environments (S1 Table). Collections were conducted either on University of Arizona property or in public lands for which nectar is not listed as requiring a collection permit (for details see S1 Table). Neither plant species is endangered or protected. We evaluated *D*. *wrightii* throughout a summer flowering season (31 May to 16 October, 2013) and *A*. *palmeri* during peak flowering in that season (14–24 July, 2013). Flowers were collected 1 h before or 16 h after anthesis (*D*. *wrightii*) or 24 h before anthesis, at anthesis, or at 24 h intervals up to 72 h after anthesis (*A*. *palmeri*). Floral visitors were allowed or were excluded from flowers with a fine mesh sleeve that excluded bats and large insects.

Flowers of *D*. *wrightii* collected in the field were processed for nectar collection within 1 h of harvesting. Nectar collection from *A*. *palmeri* flowers was performed directly on site. To collect nectar from *D*. *wrightii* we sliced each flower longitudinally and used a sterile syringe to remove all available nectar (ca. 20 uL/flower). Nectar from *A*. *palmeri* flowers was collected directly with a sterile syringe (ca. 50 uL/flower).

Each nectar sample was diluted twice (1:10 each time) and then partitioned for plating on four media (Saboraud’s agar; lysogeny broth agar, LBA; yeast potato dextrose agar, YPDA; malt extract agar, MEA). All media were prepared from standard products according to the manufacturer’s instructions (Fisher Scientific). Plates were incubated for 70 h under laboratory conditions. We then counted the number of colony forming units (CFU) per plate. Values were log-transformed for analysis and were compared by ANOVA (analyses of environment, anthesis, and floral visitors) or a generalized linear model (seasonality for *D*. *wrightii*). We measured sucrose content for flowers that had a large enough nectar volume to support microbial sampling as well as sucrose measurement (65 flowers representing six individuals of *A*. *palmeri*, and 116 flowers representing 12 individuals of *D*. *wrightii*, ecnompassing representative flowers in all study sites and throughout the duration of the study) (S1 Table).

### Characterization of microbial isolates

Representatives of each morphotype observed in each nectar sample were vouchered at the Robert L. Gilbertson Mycological Herbarium at the University of Arizona (accession numbers are listed in S1 Table). DNA was extracted from a fresh culture of each isolate following [25], with sampling of morphotypes proportional to their occurrence. To characterize bacteria we used primers 27F and 1492R to amplify a ca. 1400 basepair fragment of the 16S ribosomal RNA (16S rRNA) as described in [26]. To characterize fungi we used primers ITS1F and LR3 to amplify a ca. 1200 bp fragment consisting of the nuclear ribosomal internal transcribed spacers, 5.8S region, and the first ca. 600 bp of the nuclear ribosomal large subunit (ITS-partial LSUrDNA). PCR conditions are described in [26]. Products were evaluated by electrophoresis on a 1% agarose gel. Positive products were cleaned with Exo-SAP-IT and diluted 1:1 with molecular grade water prior to bidirectional sequencing with the above primers (5 uM) on an Applied Biosystems AB3730XL (Foster City, CA) at the University of Arizona Genetics Core. Sequences were edited and assembled as described in [27]. Consensus sequences for each isolate were submitted to GenBank (accessions KJ543743—KJ544084). The final data set consisted of 270 isolates from *D*. *wrightii* nectar (of which 210 were bacteria) and 76 isolates from *A*. *palmeri* nectar (of which 60 were bacteria).

### Operational taxonomic units and taxonomic placement

Operational taxonomic units (OTU) were designated at four levels of sequence similarity (95%, 97%, 99%, and 100%; S1 Table) in Sequencher 5.0 as described by [28]. Each sequence was compared by BLAST against the NCBI GenBank database to estimate taxonomic placement at the genus level and above. Each match was scrutinized to avoid spurious identification. We used OTU records at 97% similarity (bacteria) and 95% similarity (fungi) to estimate richness, diversity, and taxonomic composition [25–29]. We evaluated the completeness of sampling by constructing species accumulation curves in EstimateS v. 8.0 (http://viceroy.eeb.uconn.edu/estimates/). Diversity was calculated as Fisher’s alpha, which is robust to variation in sample size [29]. We compared communities of microbes in nectar via analyses of similarity (ANOSIM) in PAST (https://folk.uio.no/ohammer/past) with 999 permutations and similarity defined by the Jaccard index. Only non-singleton OTU were included. Stress values were ≤ 0.20 in each analysis. Results were visualized by non-metric multidimensional scaling (NMDS).

## Results

Microbes were found frequently in nectar of *D*. *wrightii* and *A*. *palmeri* flowers. Microbes were isolated on all four media. Bacteria were isolated most frequently on LBA and least frequently on MEA and Sabouraud’s agar (S1 Table). Fungi were isolated most frequently on Sabouraud’s agar and least frequently on LBA (S1 Table). These patterns were consistent for samples from both plant species (S1 Table).

### Nectar-inhabiting microbes of *D*. *wrightii*

**Fig 1:**
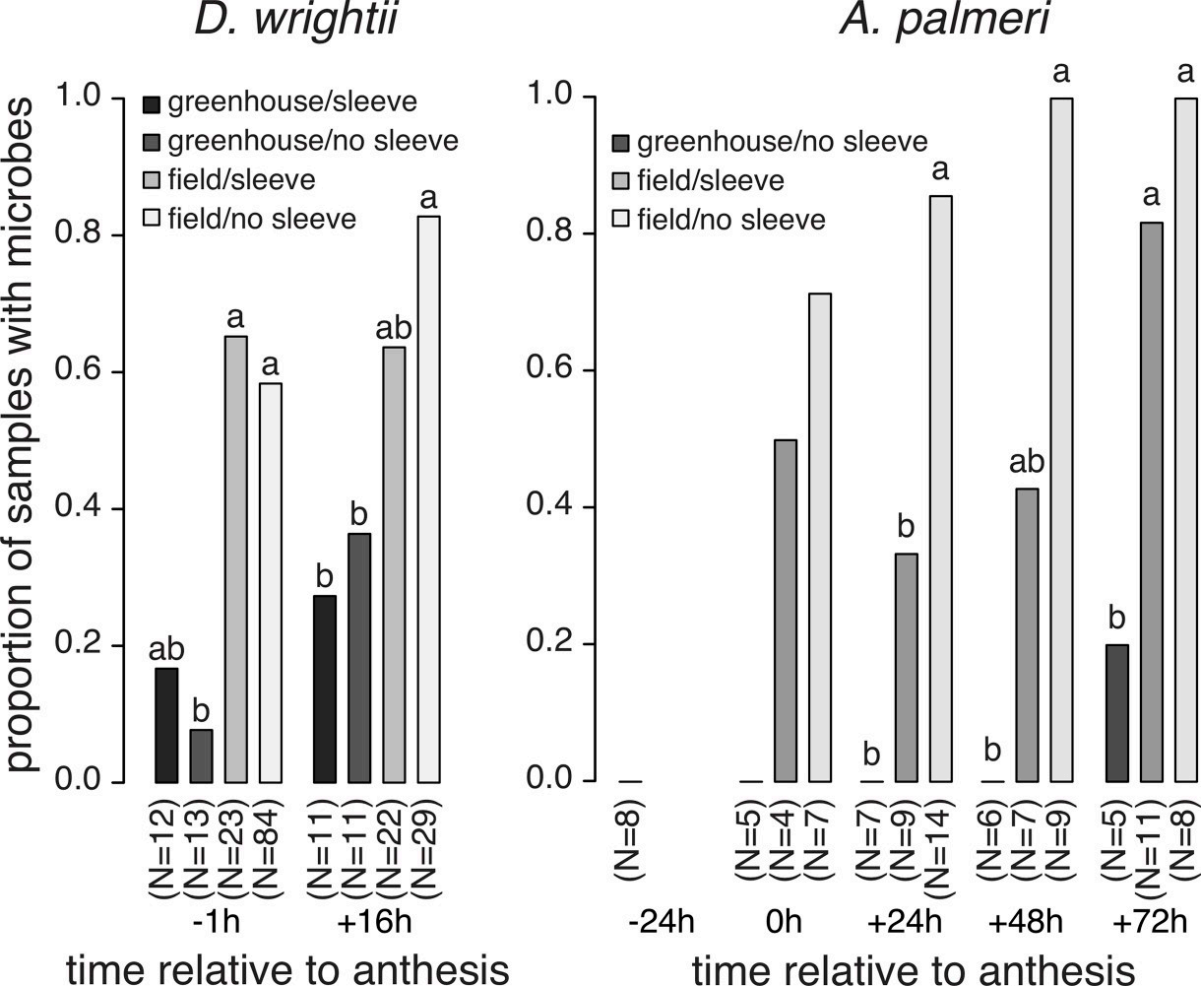
Proportion of *D*. *wrightii* and *A*. *palmeri* flowers containing nectar microbes (bacteria and fungi; one nectar sample per flower). Nectar was collected from plants in field or greenhouse environments, at different time points relative to anthesis, and with or without exclusion of flower visitors ("sleeve", "no sleeve"). Different letters assigned within a time category indicate statistically significant differences (Tukey HSD, p < 0.05).

**Fig 2:**
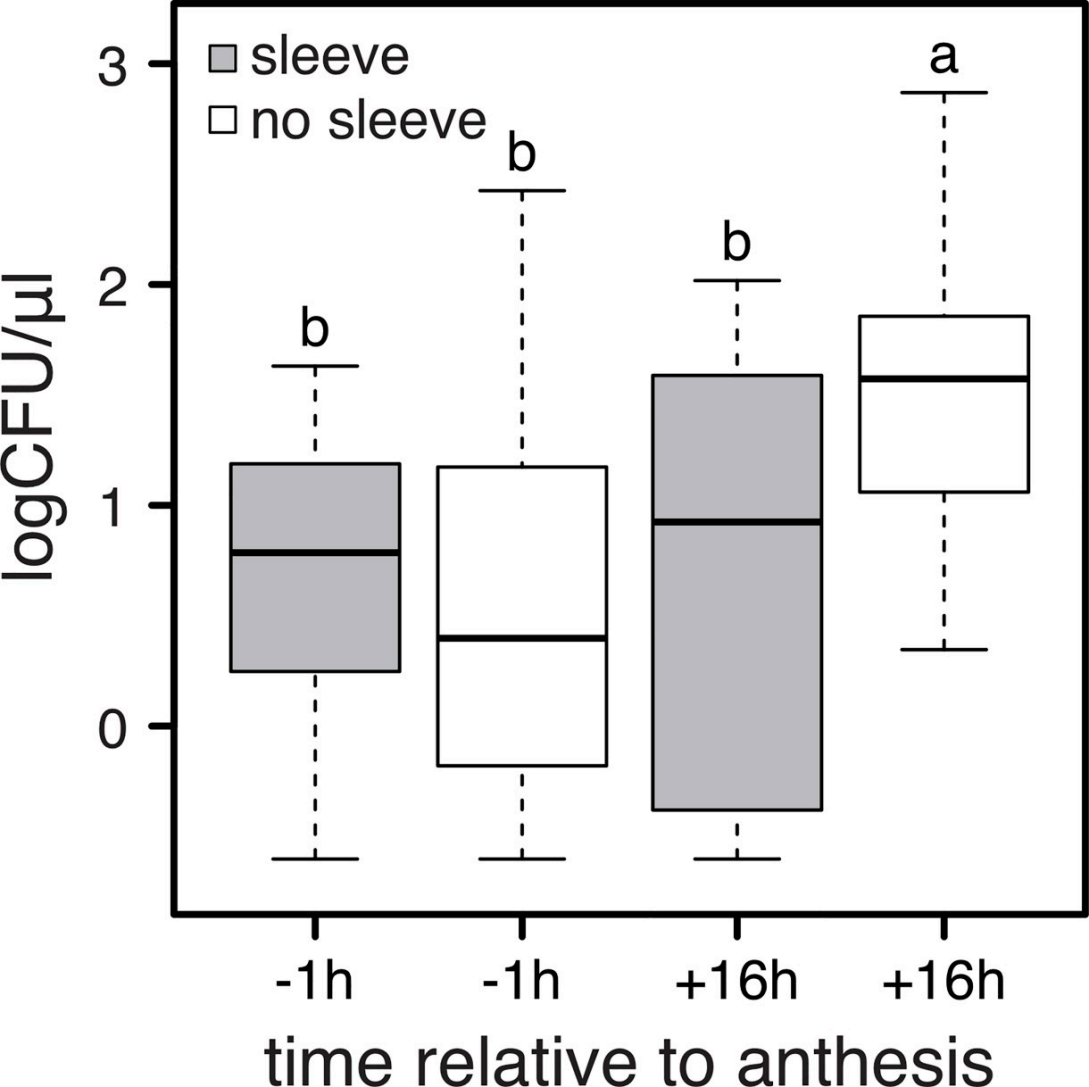
Concentration of colony forming units (CFU) in *D*. *wrightii* nectar collected before and after anthesis from plants with and without flower visitor exclusion (N = 14, 47, 12, 24 for -1 h/sleeve, -1 h/no sleeve, +16 h/sleeve and +16 h/no sleeve, respectively). Different letters indicate significant differences (Tukey HSD, p < 0.05).

**Fig 3:**
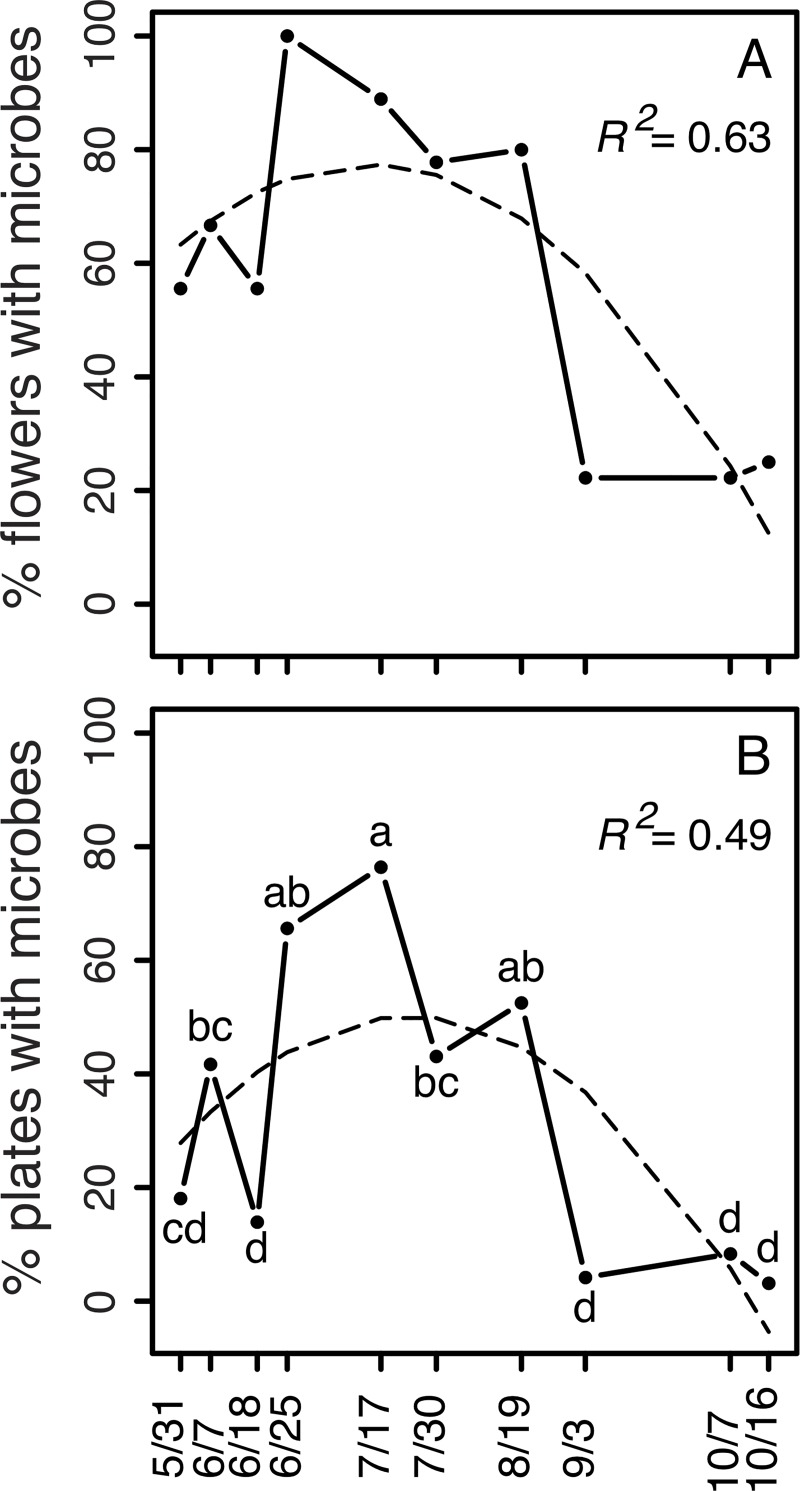
Change in abundance of nectar microbes in *D*. *wrightii* flowers during one flowering season (31 May (5/31)– 16 October (10/16), 2013). Nectar was collected from plants in the field 1 h before anthesis and without exclusion of flower visitors. (A) Proportion of *D*. *wrightii* nectar samples with microbes (N = 9 each, except for 25 June, 19 August and 16 October, with N = 8, 5, and 8, respectively). (B) Proportion of agar plates showing microbe growth (N = 72 each, except for 25 June, 19 August and 16 October, with N = 64, 40, and 64, respectively). Dashed lines describe second-order polynomial regressions. Different letters indicate significant differences between collection dates (GLM, p < 0.05).

Microbes were isolated from *D*. *wrightii* flowers under field- and greenhouse conditions, before and after anthesis, and in the presence and absence of floral visitors (Fig 1). Bacteria were isolated from *D*. *wrightii* nectar more frequently than fungi (77.8% of isolates were bacteria). The frequency of nectar samples containing microbes was greater in field conditions than in greenhouse conditions and generally was higher after anthesis than before anthesis (Fig 1). Overall, the abundance of microbes per nectar sample (i.e., CFU/uL) was greatest in flowers after anthesis that were open to floral visitors (Fig 2). Exclusion of visitors resulted in abundances of microbes after anthesis that were similar to those in flowers before anthesis (Fig 2). Overall, the percent of nectar samples and agar plates showing evidence of microbial growth was greatest in the middle of the flowering season (mid-July to mid-August) relative to earlier or later in the season (Fig 3).

Composition of bacterial communities in nectar differed marginally as a function of growing environment and floral visitation after anthesis, but did not differ between flowers before or after anthesis when pollinators were excluded, or as a function of pollinator visitation before flowers opened (Table 1). In contrast, composition of fungal communities in nectar samples differed in flowers before vs. after anthesis and, after anthesis, as a function of flower visitation (Table 1). Taxonomic affinities of representative bacteria and all fungi from *D*. *wrightii* nectar are shown in Table 2. The full list of isolates is presented in S1 Table. Before anthesis, *Pseudozyma*, *Rosenbergiella*, and *Micrococcus* were particularly common in *D*. *wrightii* nectar (S1 Table). *Candida* was isolated frequently from nectar after anthesis, but the bacterial community became diverse after anthesis (S1 Table).

**Table 1:** Effects of growing environment, age and flower visitors on nectar microbe community composition in *Datura wrightii* and *Agave palmeri* in southeastern Arizona.

Factor	group_1_	N_1_	group_2_	N_2_	R^*a*^	*p*^*a*^
**Bacteria, *D*. *wrightii***						
growing environment	greenhouse	7	field	67	0.088	0.068
age (no sleeve only)^*b*^	before anthesis^*d*^	31	after anthesis^*e*^	21	-0.004	0.510
age (sleeve only)^*c*^	before anthesis	6	after anthesis	10	0.052	0.190
flower visitors (before anthesis)	no sleeve	31	sleeve	6	-0.006	0.510
flower visitors (after anthesis)	no sleeve	21	sleeve	10	0.07	0.093
**Fungi, *D*. *wrightii***						
age (no sleeve only)	before anthesis	13	after anthesis	12	0.15	**0.008**
flower visitors (before anthesis)	no sleeve	13	sleeve	8	0.062	0.160
flower visitors (after anthesis)	no sleeve	12	sleeve	6	0.56	**0.001**
**Bacteria, *A*. *palmeri***^***f***^						
flower visitors	no sleeve	26	sleeve	5	0.056	0.220

^*a*^ Calculated with one-way ANOSIM, with Jaccard's index.

^*b*^ "no sleeve only" = included only nectar samples from flowers with no flower visitor exclusion.

^*c*^ "sleeve only" = included only nectar samples from flowers with flower visitor exclusion.

^*d*^ "before anthesis" = nectar samples collected 1 h before anthesis.

^*e*^ "after anthesis" = nectar samples collected 16 h after anthesis.

^f^ fungi from *A*. *palmeri* were not included because only 16 isolates were obtained, most of which represented OTU that occurred only one time.

**Table 2:** Top BLAST matches (genera) for the most common bacteria and all fungi isolated from nectar of *D*. *wrightii* and *A*. *palmeri* (see S1 Table for full list and details). Taxa are presented in decreasing order of abundance as isolated from *D*. *wrightii*. (%) = isolates relative to total isolates of bacteria (above) or fungi (below).

**Top BLAST match**	***D*. *wrightii*: isolates (%)**	***A*. *palmeri*: isolates (%)**
**Bacteria**		
*Rosenbergiella* sp.	21 (10.0)	13 (21.7)
*Pseudomonas* sp.	18 (8.6)	1 (1.7)
*Pantoea* and *Erwinia* sp.	15 (7.1)	4 (6.7)
*Enterobacter* sp.	15 (7.1)	6 (10.0)
*Micrococcus* sp.	14 (6.7)	1 (1.7)
*Staphylococcus* sp.	14 (6.7)	1 (1.7)
*Kocuria* sp.	11 (5.2)	3 (5.0)
*Pluralibacter* sp.	8 (3.8)	0 (0)
*Paenibacillus* sp.	7 (3.3)	0 (0)
*Bacillus* sp.	7 (3.3)	2 (3.3)
*Cronobacter* sp.	6 (2.9)	0 (0)
*Klebsiella* sp.	6 (2.9)	0 (0)
*Serratia* sp.	6 (2.9)	0 (0)
*Lactobacillus* sp.	5 (2.4)	1 (1.7)
*Acinetobacter* sp.	4 (1.9)	6 (10.0)
*Enterococcus* sp.	1 (0.5)	5 (8.3)
**Fungi**		
*Pseudozyma* sp.	31 (51.7)	0 (0)
*Candida* sp.	12 (20.0)	0 (0)
*Cryptococcus* sp.	4 (6.7)	0 (0)
*Wickerhamiella* sp.	4 (6.7)	0 (0)
*Alternaria* sp.	2 (3.3)	2 (12.5)
*Cladosporium* sp.	2 (3.3)	1 (6.3)
*Kodamaea* sp.	2 (3.3)	0 (0)
*Leptosphaeria* sp.	1 (1.7)	0 (0)
*Metschnikowia* sp.	1 (1.7)	0 (0)
*Naganishia* sp.	1 (1.7)	0 (0)
*Aspergillus* sp.	0 (0)	1 (6.3)
*Aureobasidium* sp.	0 (0)	4 (25.0)
*Clavispora* sp.	0 (0)	1 (6.3)
*Diatrypella* sp.	0 (0)	1 (6.3)
*Fusarium* sp.	0 (0)	1 (6.3)
*Thyronectria* sp.	0 (0)	5 (31.3)

### Nectar-inhabiting microbes of *A*. *palmeri*

**Fig 4:**
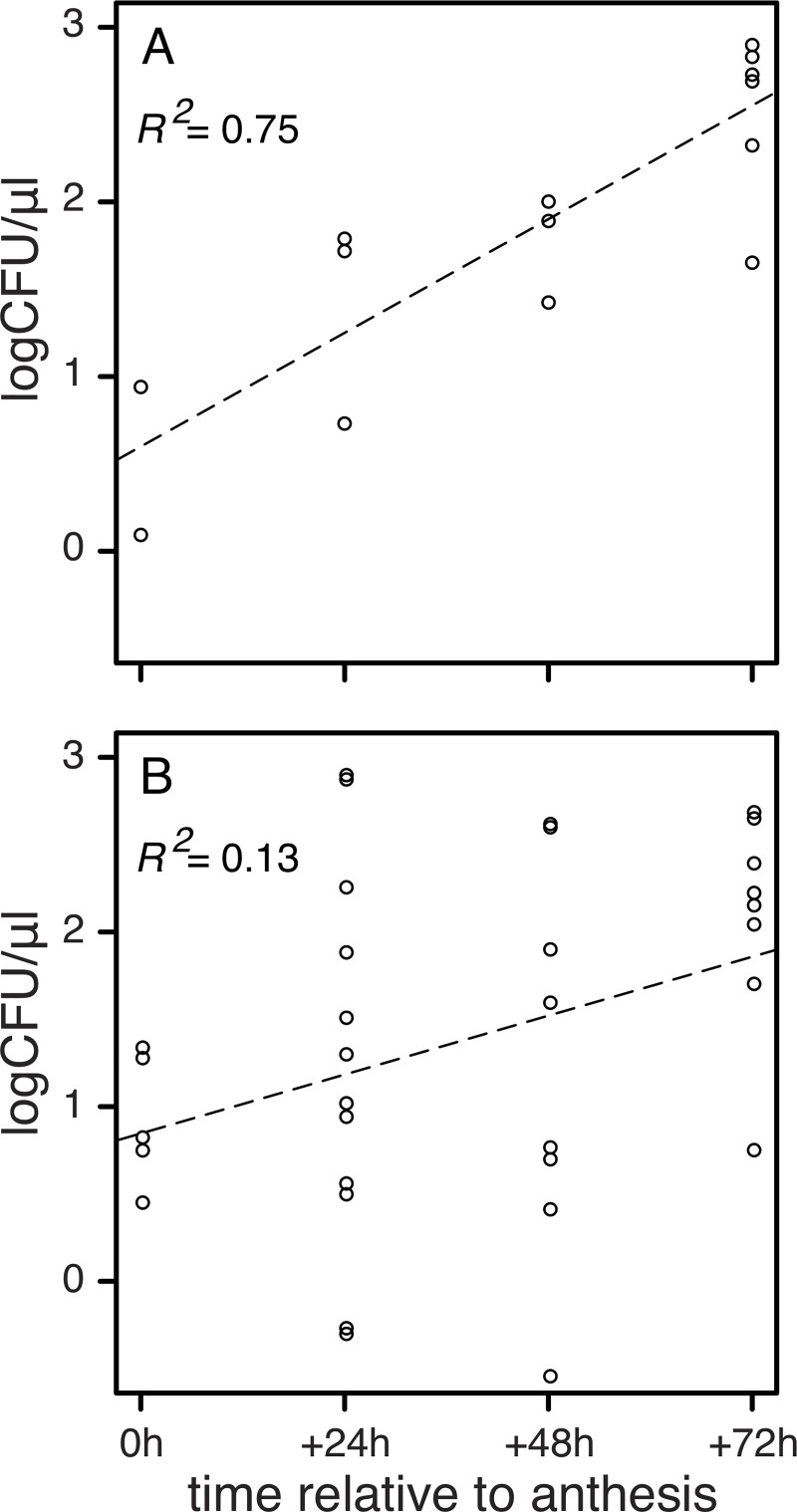
Concentration of colony forming units in *A*. *palmeri* nectar. (A) CFU concentration in the nectar of 0, 1, 2, and 3 d old *A*. *palmeri* flowers with flower visitor exclusion (N = 2, 3, 3, and 7, respectively). (B) CFU concentration in the nectar of 0, 1, 2, and 3 d old *A*. *palmeri* flowers without flower visitor exclusion (N = 5, 12, 8, and 8, respectively).

**Fig 5:**
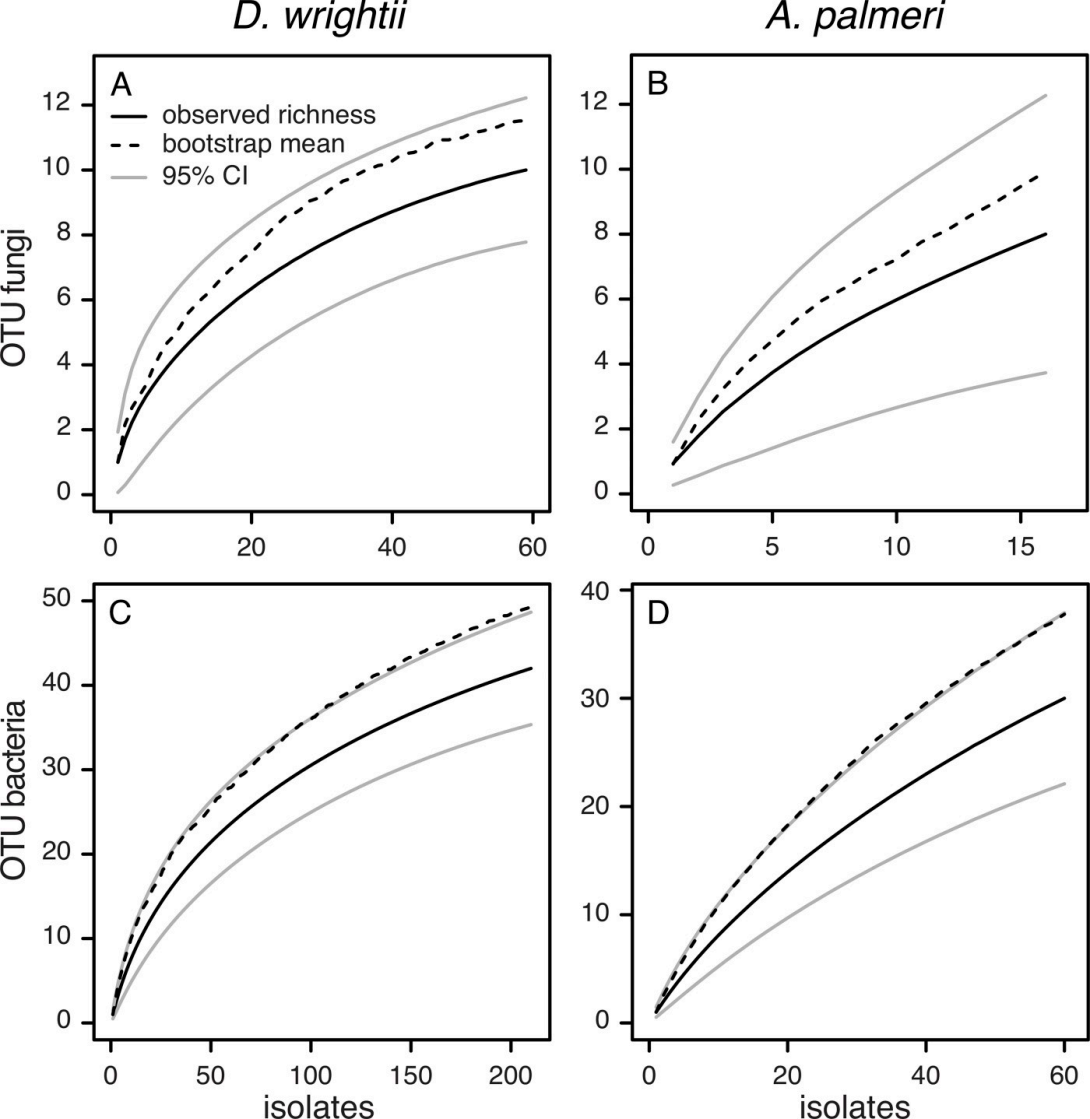
Richness of nectar microbes in *D*. *wrightii* and *A*. *palmeri* flowers. (A) Species accumulation curve for fungi in *D*. *wrightii* nectar samples (N = 59 isolates). (B) Species accumulation curve for fungi in *A*. *palmeri* nectar samples (N = 16 isolates). (C) Species accumulation curve for bacteria in *D*. *wrightii* nectar samples (N = 210 isolates). (D) Species accumulation curve for bacteria in *A*. *palmeri* nectar samples (N = 60 isolates). Figures show the number of fungi and bacteria species observed (here estimated as OTU) (Mao Tau; black lines), lower and upper 95% confidence intervals (light gray lines), and bootstrap estimate of richness (dashed lines).

Microbes were isolated from *A*. *palmeri* flowers at and after anthesis, but they were very rare before anthesis (Fig 1). Bacteria were isolated more frequently from *A*. *palmeri* nectar than fungi (76.7% of isolates were bacteria). Nectar-inhabiting microbes were more common in flowers of field-grown vs. greenhouse-grown plants (Fig 1). Flowers that were exposed to floral visitors tended to harbor more microbes than flowers from which visitors were excluded (Fig 4). The abundance of microbes per sample increased in abundance over time for flowers from which visitors were excluded (Fig 5). This increase was not as pronounced in flowers that were accessible to visitors (Fig 5). Overall, we observed no shift in bacterial communities as a function of floral visitation (Table 1). The taxonomic affinities of bacteria and fungi from *A*. *palmeri* are shown in Table 2. The full list of isolates is presented in S1 Table. We did not observe strong dominance by particular fungi or bacteria before vs. after anthesis (S1 Table).

### Comparison of nectar-inhabiting microbes of *D*. *wrightii* and *A*. *palmeri*

**Fig 6:**
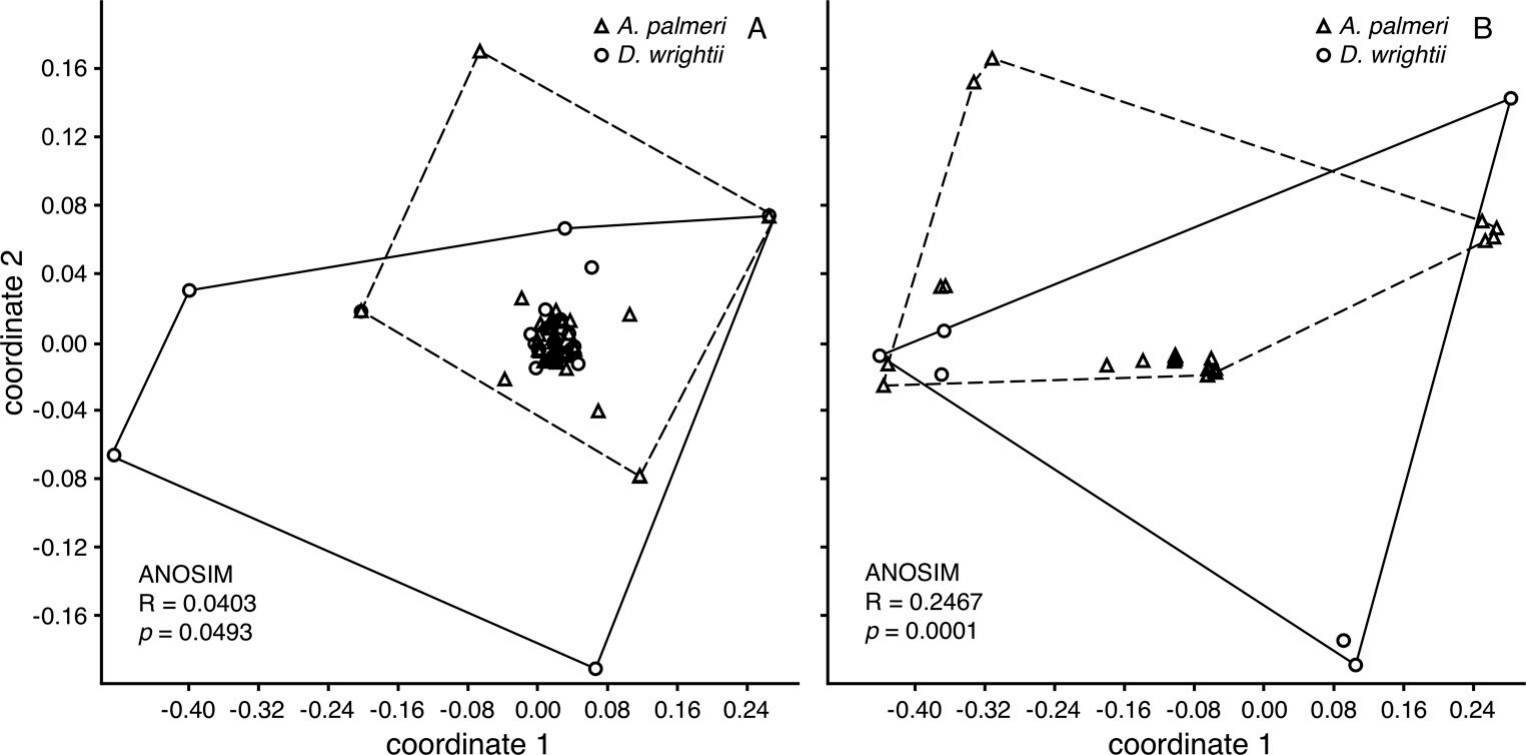
Community analysis of nectar microbe communities for *D*. *wrightii* and *A*. *palmeri*. Figure shows the results of non-metric multidimensional scaling based on Jaccard's index computed with non-singleton OTU only, and ANOSIM results for (A) bacterial (N = 31 and 47 for *A*. *palmeri* and *D*. *wrightii*, respectively) and (B) for fungal communities (N = 8 and 39 for *A*. *palmeri* and *D*. *wrightii*, respectively).

The species richness of microbes isolated from nectar of *D*. *wrightii* generally was similar to that observed in *A*. *palmeri* (Fig 5). Although the most common bacterial OTU were found in both species (Table 2), the overall composition of bacterial communities (Fig 6A) and fungal communities (Fig 6B) differed between the two plant species. The bacteria that were found in both plants species included ubiquitous genera such as *Pantoea/Erwinia* and the nectar-inhabiting genus *Rosenbergiella* [30]. Two yeasts made up >70% of the fungal isolates from *D*. *wrightii* nectar, whereas two filamentous fungi (one with a yeast form, *Aureobasidium*) comprised >50% of the fungal isolates from *A*. *palmeri* (Table 2).

## Discussion

In their review of biotic interactions between plants and other organisms in the Sonoran Desert, [9] described plant-microbe interactions as one of the largest gaps in knowledge of the regional flora and its dynamics. This study contributes to filling that gap by documenting nectar-inhabiting microbes in two night-blooming plant species for which pollination biology has been characterized previously [15–17, 31–33]. This is the first systematic survey of nectar microbiomes in the region and it provides a perspective on the microbial assemblages in ephemeral flowers of perennial plants. The culture collection and temporal perspectives generated by this study provide a basis for future work regarding how such microbes may influence nectar quality, pollinator nutrition, and pollinator specialization [1, 2, 15, 20].

In addition to carbohydrates, floral nectars also contain amino acids and fatty acids [34–37]. Hawkmoths, which forage on both plants studied here [15–17, 32], are able to use these amino acids and fatty acids for metabolic fuel or incorporate them to their somatic or reproductive tissues [38–40] with differential allocation strategies between the sexes [38]. It is possible that these amino acids and fatty acids in nectar are derived from routine senescence cycles of the nectar microbiome. If so, it is possible that pollinator foraging decisions may be related, if indirectly, to nectar microbial communities for reasons beyond carbohydrates alone.

Relative to two previous studies in the region [12, 13], the present study shows that nectar-inhabiting microbes are common and diverse at a regional level in flowers of two night-blooming species. We found that fungi and bacteria were more common in nectar of *D*. *wrightii* and *A*. *palmeri* than in the species evaluated previously [12, 13], and here highlight their abundance throughout the growing season (*D*. *wrightii*), their dynamics with respect to floral visitors (both species) and environment (greenhouse vs. field, both species), and their taxonomic composition. Our analyses reveal significant differences in the microbial communities in nectar of *D*. *wrightii* and *A*. *palmeri*, even though the most common bacterial genera occurred in both species (Table 2). Such differences may reflect many factors, including nectar chemistry and the microbiomes of pollinators. We noted that yeasts were common in nectar of *D*. *wrightii* flowers, whereas they were considered rare to absent in other plants of the region in previous work [13]. The fungal genus *Aureobasidium*, observed here in nectar of *A*. *palmeri*, appears to occur frequently in nectar of diverse plant species in other biomes [7]. In future work we anticipate examining nectar communities with culture-free methods, as these typically reveal diverse communities with members that may be recalcitrant to culturing under the methods used here [41, 42]. We also suggest that future culture-based surveys should use cultivation media that have been used in previous studies of nectar microbiomes in other plants and geographic locations [1–8, 18–24, 41–44]: such approaches will clarify the novelty and distinctiveness of microbiomes in nectar of the night-blooming plants of the Sonoran Desert as studied here. Different media fostered growth by distinctive portions of the microbiome in the present study, consistent with differences in pH and nutrient content and underscoring the importance of considering media carefully for culture-based studies of nectar communities. Commonly used media in nectar microbiome studies (e.g., trypticase soy agar [4], or R2A and yeast medium [45]) could be especially useful in future work.

Nectar microbes have been studied most extensively in plants with flowers that are receptive over multiple days, and in those cases microbes appear to colonize nectar mostly after anthesis via pollinators and airborne deposition [3, 22, 43]. Here we documented the occurrence of microbial communities in nectar before anthesis in two species, but especially in *D*. *wrightii*, which has a flower longevity of only one night. Hence, our results suggest that microbes could alter nectar composition and chemistry in the absence of pollinators. We observed that thrips were common in and on these flowers before anthesis and suspect that they may serve as vectors for movement of microbes among flowers before pollinators can access them [44]. In future work we suggest quantifying thrips on flowers after anthesis, on bagged flowers, and under different settings to understand their roles. Measuring visitation rates by pollinators and other insects to flowers also could inform our results and will be important in future work.

After anthesis, microbes generally increased in abundance in nectar, a pattern observed in flowers with and without pollinator visitation. In *D*. *wrightii* such visitation was associated with significant changes in fungal community composition, as was the transition from pre- to post-anthesis (Table 1). Our results are broadly consistent with microbial communities developing with input from pollinators but also with population growth of microbial populations in nectar over time. In general bacterial community composition was less sensitive to such factors, a topic to be explored in further work.

Although we were not able to sample nectar from *A*. *palmeri* and *D*. *wrightii* in the same location, samples for these species came primarily from the Tucson area, where both species occur as ornamentals and in small patches of native vegetation throughout the city. These species have overlapping distributions and flower concurrently in the Tucson region [15]. Removal of data from Box Canyon (*A*. *palmeri* only; ca. 30 km southeast of Tucson; S1 Table) did not change our main conclusions. The bats that pollinate *A*. *palmeri* occur in the Tucson basin and cover large distances nightly. For example it has been shown that the foraging radius of *Leptonycteris* colonies can be 30–50 km, and these bats frequently move pollen over long-distances [46]. Therefore, we do not have reason to expect a strong geographic pattern to the distribution of nectar microbes; however, further sampling to evaluate this pattern is needed. Similar studies concerning hawkmoth foraging behavior are lacking and knowledge about flight range in a natural setting is scarce, but *M*. *sexta* that forage on *A*. *palmeri* and pollinate *D*. *wrightii* are numerous in Tucson and were observed commonly at all of our study sites. In a laboratory setting it has been shown that *M*. *sexta* can cover distances of several kilometers (5.8 ± 2.7 km) within 3 h [47], which indicates that they can act as long-distance pollen dispersers as well and encounter different *D*. *wrightii* and *A*. *palmeri* populations during foraging bouts.

The Sonoran Desert is known for its iconic mutualisms between plants and pollinators [9, 10]. In this biodiverse region, both flowering plants and their native pollinators are diverse, frequently endemic, and often threatened by human activity and climate shifts. Our long-term aim is to integrate a perspective based on microbes to help understand the dynamics of such mutualisms, their ecological traits, and their evolution, and the cryptic ways in which they are sensitive to anthropogenic activities at local and regional scales.

## Supporting information

S1 Table Data regarding microbial isolates. (XLSX)
